# Usefulness of Tissue Doppler Imaging for the Evaluation of Pulmonary Hypertension in Canine Heartworm Disease

**DOI:** 10.3390/ani13233647

**Published:** 2023-11-25

**Authors:** Jorge Isidoro Matos, Sara Nieves García-Rodríguez, Noelia Costa-Rodríguez, Alicia Caro-Vadillo, Elena Carretón, José Alberto Montoya-Alonso

**Affiliations:** 1 Internal Medicine, Faculty of Veterinary Medicine, Research Institute of Biomedical and Health Sciences (IUIBS), University of Las Palmas de Gran Canaria, 35016 Las Palmas de Gran Canaria, Spain; jorge.matos@ulpgc.es (J.I.M.); saranieves.garcia@ulpgc.es (S.N.G.-R.); noelia.costa@ulpgc.es (N.C.-R.); aliciac@vet.ucm.es (A.C.-V.); alberto.montoya@ulpgc.es (J.A.M.-A.); 2 Hospital Clínico Veterinario, Faculty of Veterinary Medicine, Universidad Complutense de Madrid (UCM), 28040 Madrid, Spain

**Keywords:** heartworm, *Dirofilaria immitis*, pulmonary hypertension, echocardiography, tissue Doppler imaging

## Abstract

**Simple Summary:**

Tissue Doppler imaging is a useful echocardiographic technique to evaluate the systolic and diastolic function of the right ventricle and has been used as an estimator of pulmonary hypertension. The objective of this study was to evaluate the diagnostic value of tissue Doppler echocardiography to determine the presence of pulmonary hypertension in dogs with heartworm disease. A total of 116 heartworm-infected dogs and 33 healthy dogs were included in the study. Echocardiographic determinations were performed following established protocols to determine the presence of pulmonary hypertension in 47.4% of dogs infected with heartworm. Subsequently, a total of eight echocardiographic measurements were performed using tissue Doppler imaging to determine their usefulness in the diagnosis of pulmonary hypertension. Overall, the results showed significant differences with respect to the presence or absence of pulmonary hypertension. Furthermore, acceptable cut-off values were reported to estimate the presence of pulmonary hypertension in most echocardiographic measurements determined using tissue Doppler imaging. The measures analysed have demonstrated their usefulness as a complementary tool to determine pulmonary hypertension in dogs with *Dirofilaria immitis*.

**Abstract:**

Background: *Dirofilaria immitis* is a nematode that produces proliferative pulmonary endarteritis in dogs due to direct contact of the adult parasites with the intima layer of the pulmonary arteries, leading to irreversible severe structural damage and sustained pulmonary hypertension (PH), which can produce severe cardiorespiratory disorders. The purpose of this study was to assess the diagnostic value of the echocardiography tissue Doppler imaging (TDI) in determining the presence of PH in dogs with heartworm disease. Methods: There were 116 heartworm-infected dogs with PH and 33 healthy dogs included in the study. Based on the right pulmonary artery distensibility index (RPADi) < 29.5%, PH was present in 47.4% of infected dogs. Additionally, the animals were evaluated using other standard alternative echocardiographic measures to estimate PH. Moreover, a total of eight echocardiographic measurements were analysed using the TDI to determine its usefulness in diagnosing PH (E′, A′, S, E′:A′, global TDI, HRI-IVCT, HRI-IVRT, R-TEI). Results: The TDI measurements showed significant differences between dogs with and without PH, demonstrating a positive correlation with respect to the RPADi. In addition, cut-off values for the detection of PH with excellent sensitivity and specificity were found for E′:A′, global TDI, HRI-IVCT, HRI-IVRT and R-TEI. Conclusions: The TDI mode may be useful as an adjunct diagnostic method for the determination of PH in dogs with *Dirofilaria immitis.*

## 1. Introduction

Heartworm disease is a parasitic disease caused by *Dirofilaria immitis* (Leidy, 1856), found worldwide and mainly affecting domestic and wild canids and felids. Heartworm disease produces severe cardiorespiratory disorders in dogs which require significant economic investment in endemic areas where regular prophylactic measures must be performed [1]. Chronic contact of the adult parasite of *D. immitis* with the intimal layer of the pulmonary vessel causes proliferative endarteritis, inducing dilatation, loss of lumen and loss of elasticity. Obstruction of blood flow can also occur, raising the pressure in the pulmonary arteries due to increased pulmonary vascular resistance. This increase is known as precapillary pulmonary hypertension (PH) [2], responsible for most of the clinical signs observed in dogs infected by *D. immitis*. Chronically, PH can cause hypertrophy and dilation of the right ventricle in its attempt to compensate for lung perfusion complications, leading to fatal right heart failure [3].

Nowadays, echocardiography sets the best technique for the detection of PH in daily veterinary practice, due to its accuracy and convenience [2]. To determine PH caused by *D. immitis*, previous research has determined that the right pulmonary artery distensibility index (RPADi) is the most accurate non-invasive measure to estimate the presence of increased pulmonary artery pressure, especially when tricuspid regurgitation velocity and pulmonary regurgitation are not evaluable [4,5,6].

Tissue Doppler imaging (TDI) is an echocardiographic method focused on the analysis of segmental myocardial movements in the systolic and diastolic phase. The degree of movement is determined by the cardiac contractility, the volume of the cardiac chambers and pressure differences, and allows for analysis of the systolic and diastolic functions [7]. Currently, most of the published studies have considered the use of TDI as a fundamental echocardiographic mode for analysing cardiac ventricular function and its use has been standardised in routine cardiac assessment [8,9]. A few studies have demonstrated the usefulness of TDI in estimating right ventricular dysfunction caused by PH in dogs, and have studied the diagnostic value of TDI in canine patients with PH caused by several conditions [10,11,12,13,14]. These studies concluded that TDI measurements of the systolic and diastolic phases of the right heart acted as an effective tool to estimate PH because a slight increase in pulmonary artery pressure alters the systolic and diastolic myocardial functions in the right heart. To date, the diagnostic value of TDI in the evaluation of PH has focused mainly on post-capillary causes in small animals, in which the changes originate from abnormalities in the left heart structures such as myxomatous mitral valve disease, canine dilated cardiomyopathy or feline hypertrophic cardiomyopathy [10,11,12,13,14,15,16,17]. Therefore, the use of TDI in heartworm disease has not been previously reported in dogs.

The haemodynamic changes that can be detected using TDI in the right heart chambers, caused by the presence of adult parasites, might be a useful tool for the evaluation of PH in canine heartworm disease. Thus, the aim of this study was to assess the utility of the measurements obtained via echocardiographic examination in TDI mode (E′, A′, S, E′:A′, global TDI, HRI-IVCT, HRI-IVRT, R-TEI) to detect the presence of PH in dogs infected by *D. immitis*.

## 2. Materials and Methods

### 2.1. Studied Animals

This prospective study included 149 client-owned dogs that visited the Veterinary Teaching Hospital of the University of Las Palmas de Gran Canaria (Canary Islands, Spain) between September 2021 and July 2022 and lived in a hyperendemic area of *D. immitis* [18]. A complete record was kept for each animal, including identification (age, sex, breed and weight), determination of body condition score (BCS) [19], as well as evaluation of the presence/absence of respiratory symptoms (i.e., cough, dyspnoea, exercise intolerance) and signs of right-sided congestive heart failure (R-CHF) mainly based on evidence of ascites, pleural effusion, jugular pulse and cava vein distention.

The presence/absence of heartworm was diagnosed by the detection of circulating *D. immitis* antigens, using a commercial kit (Uranotest Dirofilaria^®^, Urano Vet SL, Barcelona, Spain). Dogs with a negative test were used as healthy control animals, based on history, physical examination, cardiovascular evaluation and echocardiographic exam. In all cases, dogs with evidence of cardiac conditions coexisting with heartworm disease or dogs that had previously received any cardiovascular medication were excluded from the study.

### 2.2. Echocardiography

Echocardiographic exams were performed with the animals conscious and restrained without the use of any sedation, using an ultrasound equipment with spectral and colour Doppler and multifrequency phased-array transducers (2.5–12 MHz, Viviq Iq^®^, General Electric, Boston, MA, USA). Electrocardiographic monitoring was performed in all patients. Dogs were placed in right and left lateral decubitus with the transducer placed in the third-fourth intercostal space according to the described and validated methods [7,8]. Three consecutive cardiac cycles were performed to determine each of the measurements studied. All echocardiographic examinations were performed by the same researcher.

The presence or absence of PH was determined in accordance with the guidelines of the American College of Veterinary Internal Medicine (ACVIM) (REF). The RPADi and the tricuspid regurgitation systolic flow, among other measures taken routinely, were used as previously described [2,4,20,21]. The animals were classified with the presence of moderate/severe PH when they presented an RPADi < 29.5%, a tricuspid regurgitation systolic flow > 3.4 m/s, as well as other standard echocardiographic indicators ensuring the presence/absence of PH, as described below [21,22,23,24,25]. The pulmonary-vein-to-pulmonary-artery ratio in a right parasternal long axis view (PV:PA ratio) and the relation between the pulmonary trunk and ascending aorta taken in a right parasternal shot axis view (PT:Ao) were recorded. The right ventricular outflow tract velocity was measured, and the relationship between the acceleration times (AT) and ejection times (ET) was determined using the AT:ET ratio. The peak of the tricuspid regurgitation velocity was also measured to obtain the tricuspid regurgitation pressure gradient (TRPG), along with the systolic displacement of the tricuspid annulus (TAPSE).

**Figure 1:**
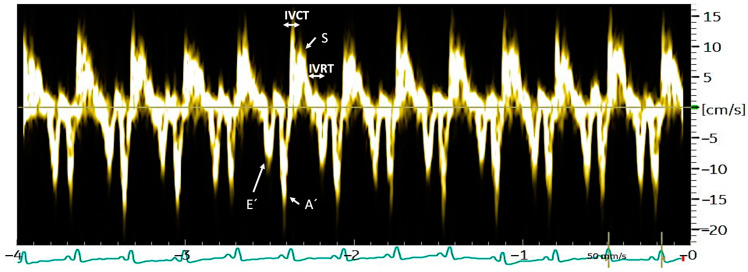
Measurement of the TDI parameters E′, A′, S, HRI-IVCT and HRI-IVRT in a dog with heartworm disease that presented pulmonary hypertension (right pulmonary artery distensibility index < 29.5%). Left parasternal position, apical four-chamber, longitudinal section in TDI mode, focusing on the tricuspid valve annulus.

Right TDI examinations were performed as previously described and validated [7,10] (Figure 1). Longitudinal velocities were measured in a basal segment of the mid-inner portion of the right myocardium, focusing on the tricuspid valve annulus, using a longitudinal four-chamber left apical view optimised for the visualisation of the right ventricle and atrium [10]. The maximum myocardial velocities were measured in early diastole (E′), late diastole (A′), and systole (S), calculating the E′:A′ ratio. A global TDI index of the myocardial function (global TDI), based on the formula global TDI = S × E′:A′, was also calculated.

The RR interval was established on the same 3 consecutive cardiac cycles due to the significance of heart rate in the measurements. Therefore, TDI time indices were indexed to the RR interval, defining heart-rate-indexed (HRI) time indices, as previously published [9,10]. Myocardial time indices were measured, including the isovolumic contraction time (HRI-IVCT) and isovolumic relaxation time (HRI-IVRT). The HRI-IVCT was defined as the time between the end of the A′ wave and the beginning of the S wave and the HRI-IVRT as the time between the end of the S wave and the beginning of the E′ wave. The R-TEI index was calculated using the following formula (HRI-IVRT + HRI-IVCT]/ET) [23,24].

Finally, the presence or absence of worms in the pulmonary arteries and right heart chambers was evaluated, and the parasite burden was classified as low or high, according to the guidelines previously published [26].

Based on the RPADi and clinical status, dogs were classified into 3 groups. Group A comprised healthy control animals, Group B included heartworm-infected dogs without PH and group C was formed of heartworm-infected dogs with PH.

### 2.3. Statistical Analysis

Frequencies and percentages were shown for categorical variables. The differences in parameters between groups were evaluated using Pearson’s non-parametric Chi^2^ test and, only in the case of 2 × 2 tables, Fisher’s exact test was applied. For continuous variables, the differences in the parameters between groups were evaluated by means of Mann–Whitney/Kruskall–Wallis tests (non-parametric) or T-student/ANOVA (parametric) based on the normality of the variables to be evaluated by means of a Shapiro–Wilks test. To establish the correlation and explanatory capacity of the methods with the gold standard (RPADi), linear regression was applied and the non-standardised beta coefficients, their 95% CI, the R^2^ and the equation were shown. To determine the ability of the different echocardiographic measurements to classify the presence/absence of PH, the receiver operating characteristic (ROC) curve was used. All multiple comparisons were adjusted using the Bonferroni correction. All the contrasts were accompanied by the effect size estimator to complete the interpretation of the results. For categorical variables, it was Cramer’s V and, for continuous variables, it was Cohen’s d. The criteria for the classification of the magnitude of the effect were as follows: Cohen’s d: small (d = 0.2–0.4), medium (d = 0.5–0.8) and large (d = greater than 0.8). Cramer’s V: 0.00–0.09 as negligible, 0.10–0.29 as low, 0.30–0.49 as medium and from 0.50 as high. For all cases, the significance level used was 5% (α = 0.05).

### 2.4. Ethical Statement

All owners were informed and gave their consent to participate in the study. Ethical approval was not required for this study, as it was a purely observational study with voluntary enrolment and did not involve additional invasive clinical diagnostic procedures. The evaluation of the study included ethical considerations and legal aspects regarding animal protection and welfare, and was conducted in accordance with the current Spanish and European legislation on animal protection.

## 3. Results

Presence of heartworm was diagnosed in 116 dogs (77.85%) while 33 (22.15%) dogs were used as healthy control animals. Based on the RPADi, all healthy dogs were normotensive (Group A, n = 33), whilst PH was absent in 52.6% of dogs infected with *D. immitis* (Group B, n = 61). Group C included 55 dogs with heartworm and presence of PH.

The results of the descriptive analytical study of the clinical variables and the standard and TDI mode echocardiographic measurements in the studied dogs are shown in Table 1. All the animals studied presented age ranges from 3 to 14 years and showed similar proportions of both sexes (50.3% females and 49.7% males). The BCS ranged from 1 to 9 [19] and the weight range varied between 3.2 and 45.9 kg. The presence of mongrel dogs was greater than the remaining breeds analysed (49.1%), with a total of 29 different breeds recorded. No significant differences in age, sex, BCS, weight or breed were reported between the groups A, B and C.

The parasite burden ranged from 1 to 4 [21,26] in the dogs of groups B and C, with no significant differences observed between them, although a higher mean value was observed in dogs from group C. The parasite load was obviously 1 (no worms visible) in dogs from group A. The presence of respiratory symptoms and signs of R-CHF were directly related to the presence of heartworm and PH, since all dogs with R-CHF occurred in group C, and respiratory symptoms increased considerably in the animals suffering from PH.

The statistical study revealed significant differences in the RPADi between group C in comparison to groups A and B (*p* = 0.000), and no differences were found between groups A and B. The results of the additional standard echocardiographic measurements to estimate the presence of PH showed significantly higher values for TRPG and PT:Ao in group C when compared with groups A and B (*p* = 0.000). There were also significantly lower values for the AT:ET ratio, TAPSE and PV:PA ratio in group C when compared with groups A and B (*p* = 0.000). However, no differences were observed in the echocardiographic parameters studied between groups A and B.

**Figure 2:**
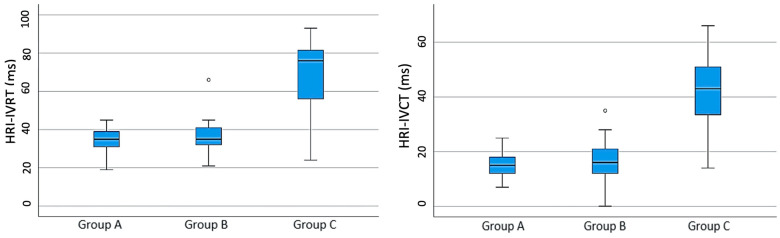
Scatter plots illustrating echocardiography TDI measurements of HRI-IVRT and HRI-IVCT obtained in dogs without heartworm infection (group A), in dogs with heartworm and without pulmonary hypertension (PH) (group B) and in dogs with heartworm and PH (group C).

Similarly, ANOVA tests found significant differences between the values of the TDI parameters in the studied groups. The Bonferroni post hoc tests for multiple comparisons indicated that, for all the parameters, except for the A′ measurement, there were significant differences in dogs from group C when compared to groups A and B. The values of E′, S, E′:A′ and global TDI were significantly lower in dogs from group C, whilst the values of HRI-IVCT, HRI-IVRT and R-TEI were significantly higher in dogs from group C (*p* = 0.000) (Figure 2). The results obtained in the A′ measurement did not report differences between the groups of animals studied (*p* = 0.166).

**Figure 3:**
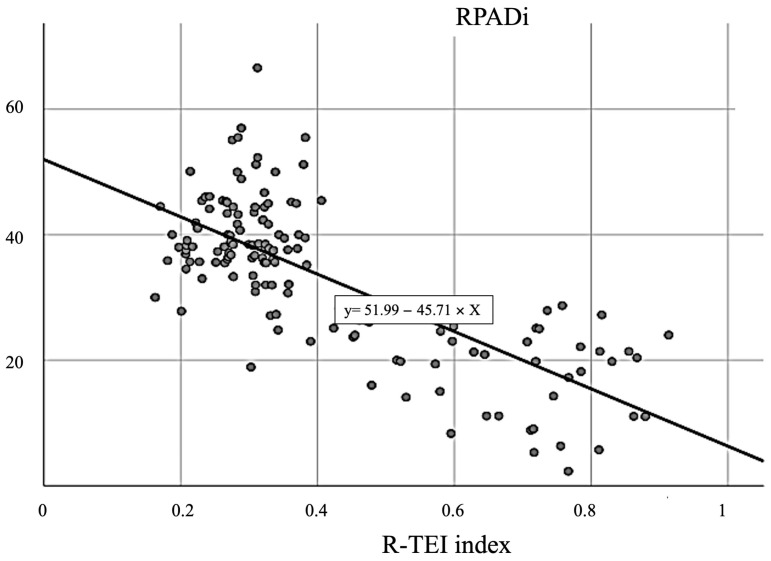
Scatter plots illustrating significant (all *p* < 0.01) correlations (R) between right pulmonary artery distensibility index (RPADi) and R-TEI index. The solid line within each scatter plot represents the line of best fit. Lineal R^2^ = 0.555.

Linear regression was carried out for each TDI parameter studied, showing that the parameters that best correlated with the RPADi were HRI-IVCT, HRI-IVRT and R-TEI, with R^2^ > 0.5 (Figure 3). The parameters E′, S, E′:A′ and global TDI had an R^2^ close to 0.3. The result obtained for A′ showed that this measurement did not correlate with the RPADi (R^2^ < 0.1).

The results of the ROC curves for each of the TDI measurements to determine the presence or absence of PH and the cut-off points of the parameters that maximise sensitivity and specificity (using the Youden index) are collected in Table 2, showing excellent areas under the curve (AUCs) (>0.9) for E′:A′, global TDI, HRI-IVCT, HRI-IVRT and R-TEI. The sensitivity and specificity of the cut-off points established for these measures for the diagnosis of PH obtained excellent results for E′:A′ (87.3%, 92.6%, respectively), HRI-IVCT (83.6% and 95.7%, respectively) and HRI-IVRT (87.3% and 98.9%), highlighting global TDI (94.5% and 98.9%, respectively) and R-TEI index (90.9% and 98.9%, respectively). The values of S and E′ showed acceptable AUCs (>0.8), while the worst model was A′ with an AUC of 0.572.

## 4. Discussion

The presence of PH is a severe hemodynamic condition that usually occurs in dogs parasitised by *D. immitis* and is responsible for multiple cardiorespiratory complications that characterise canine heartworm disease [3,4], including chronic systolic and diastolic dysfunction in the right cardiac chambers. So far, echocardiographic measurement of the RPADi has proven useful for the diagnosis and staging of PH in dogs with heartworm [5,6]. However, new non-invasive methods must be incorporated to assess in a more exact and precise way the cardiovascular repercussions of this disease. In this sense, the TDI mode is proposed as an effective complementary tool for the echocardiographic analysis of the haemodynamic repercussions in heartworm disease.

In this study, the guidelines of the ACVIM for the diagnosis of PH have been used [2]. Since some standardised measures, such as pulmonary and tricuspid regurgitation, are not always detectable [20,22], the RPADi was used as a gold-standard measure to identify the presence of PH in dogs with heartworm disease [3,4,5,6], whilst other echocardiographic parameters were used to confirm the presence of PH [23,24,25]. In the present study, the measurements of PT:Ao, AT:ET, TRPG, TAPSE and PV:PA ratio were used, which have a widely demonstrated diagnostic value in the study of canine PH caused by *D. immitis* [21,22,23]. The results for all the studied standard echocardiographic measures obtained in the present research have obtained highly similar values to those previously published in heartworm disease and confirm their usefulness in the study of this parasitic disease [5,6,21].

The TDI echocardiographic mode is widely used in veterinary medicine and its effectiveness is particularly important in providing information on systolic and diastolic function triggered by cardiovascular diseases. Previously, the use of echocardiographic measurements using the TDI mode has shown its efficacy for the assessment of volume overload and its effects on left ventricular dysfunction in dogs with myxomatous mitral valve disease and dilated cardiomyopathy [8,11,13,14,15]. Furthermore, it has demonstrated its usefulness in the echocardiographic study of other species, such as cats with hypertrophic cardiomyopathy [16,17], horses with heart disease [27] and in the evaluation of cardiac function in healthy animals such as rabbits and goats [28,29].

Regarding the detection of PH, the study of TDI mode has already demonstrated its usefulness in dogs, specifically in the diagnosis of PH in dogs mainly suffering from left heart pathologies [7,8,9]. The results of the present study agree with those from previous authors [10,11,14], who reported severe decreases in the values of E′, E′:A′ and S, as well as increases in the values of HRI-IVCT and HRI-IVRT; moreover, these authors noted excellent sensitivities and specificities for global TDI and E′:A′ (Se 89%; Sp 93% and 90%, respectively) [10], with similar cut-off points to those reported in the present study. Therefore, although the methodologies varied and other pathologies that cause PH were evaluated, the results showed the great usefulness of the TDI mode to determine the presence of PH, as seen in the present study, in which the results confirm the utility of the TDI mode to determine PH in dogs with heartworm. Thus, this method shows important value in the clinical study of infections by *D. immitis*, by demonstrating excellent correlations between TDI measurements (HRI-IVCT, HRI-IVRT and R-TEI) and the presence and severity of PH, as well as high sensitivity and specificity values when establishing cut-off points for the detection of PH for most TDI measurements. Another study, unlike the present one, found significant differences in the value of A′ in dogs with PH [11], although, again, this can be justified by the difference in the methodology and pathologies studied. In any case, the results of the present study suggest that the presence of PH can be estimated by means of TDI measurements regardless of its origin, and therefore bestows great value in being used in veterinary cardiology.

The results observed agree with the cardiovascular alterations present in canine heartworm. In this sense, the presence of adult parasites in the lumen of the pulmonary arteries generates proliferative endarteritis that causes a decrease in the distension capacity during the cardiac cycle, an increase in pulmonary arterial pressure and therefore morphological changes with loss of myocardial functionality. When this event becomes chronic, tricuspid insufficiency occurs due to the increased pressure in the right ventricle. Finally, at the final stages, signs of atrial dilation and retrograde venous congestion are generated with a decrease in the speed of passive diastolic filling (E′) and also a decrease in systolic velocity (S) due to volume overload. However, the active diastolic filling rate (A′) remains unchanged because it does not depend on pressure differences in the cardiac chambers. Likewise, it is logical that the aforementioned pressure and volume overloads cause increases in the relaxation (HRI-IVRT) and contraction (HRI-IVCT) times adjacent to ventricular systole. Similarly, measures that adjust these haemodynamic changes to analyse them more precisely, such as the global TDI and the R-TEI using diastolic and systolic measurements in TDI mode, have shown satisfactory results in the present study.

No significant differences were found according to age, sex, weight, breed or BCS between groups, coinciding with previous studies that determined that *D. immitis* infection does not show preferences regarding these epidemiological parameters, nor does the development of PH in the presence of this pathology [3,5,18,20].

A higher parasite burden was reported in animals with heartworm suffering PH, although the statistical differences were not significant. The influence of the parasitic load on the development of PH is not clear. Some authors claim that the greatest influence on the development of endarteritis and PH corresponds to the chronicity of the disease, the intensity of exercise and the immune response of the host to the parasite [4,5,26], whilst other authors attribute an important role also to the parasite load [3,30,31].

Most dogs with cardiorespiratory symptoms corresponded with those with PH; previous studies have demonstrated that approximately 50% of infected animals developed PH and, therefore, associated symptoms [4,5,6,21]. The presence of PH is a negative prognostic factor in infected dogs [2,23] and, considering the severity and frequency of PH, which also appears to be irreversible in many patients [3,4,21], it is crucial to keep developing non-invasive and affordable techniques to detect this condition in dogs with *D. immitis*.

## 5. Conclusions

The results showed that the TDI measurements of E′, S, E′:A′, HRI-IVRT, HRI-IVCT, global TDI and R-TEI differentiated between normotensive dogs and those with PH (RPADi < 29.5%). Therefore, they can be a helpful alternative for evaluating patients with heartworm via transthoracic echocardiography. Similarly, the A′ measure proved to be an inadequate indicator for estimating PH in dogs with heartworm disease. The HRI-IVCT, HRI-IVRT and R-TEI showed higher levels of efficiency in the detection of PH in dogs parasitised by *D. immitis*, although further studies are indicated with the aim of obtaining standardised protocols and exact reference values to satisfactorily establish the diagnosis and estimate the severity of PH in dogs with heartworm disease.

## Figures and Tables

**Table 1:** Clinical characteristics and echocardiography parameters of study dogs (n = 149). Data represent median unless otherwise stated. CHF: congestive heart failure; BCS: body condition score; HR: heart rate. Group A comprised healthy control animals, Group B included heartworm-infected dogs without pulmonary hypertension (PH) and group C was formed of heartworm-infected dogs with PH. RPADi: right pulmonary artery distensibility index; TRPG: tricuspid regurgitation pressure gradient; PT: pulmonary trunk; PV:PA: pulmonary vein to pulmonary artery ratio; TASPSE: tricuspid annular plane systolic excursion; AT:ET: right ventricle outflow Doppler acceleration time to ejection time ratio; HRI-IVCT: isovolumic contraction time indexed to heart rate; HRI-IVRT: isovolumic relaxation time indexed to heart rate; Global TDI: global TDI index defined as S × E′:A′; R-TEI index: Tei index of right ventricular myocardial performance.

	DOGS (N = 149)	GROUP A (N = 33)	GROUP B (N = 61)	GROUP C (N = 55)	*p*-Value
Age (years)	7.67 ± 3.50	8.51 ± 3.75	6.43 ± 2.78	8.54 ± 3.64	0.01 (0.60) ^1^
Female: number (%)	75 (50.33%)	15 (10.07%)	36 (24.16%)	24 (16.11%)	0.21 ^3^
Body weight (kg)	18.45 ± 10.34	16.44 ± 10.15	18.70 ± 10.42	19.31 ± 10.40	0.47 ^2^
BCS (1–9)	5.28 ± 0.99	5.36 ± 0.99	5.31 ± 0.77	5.20 ± 1.14	0.36 ^2^
Breed: mongrel (%)	75 (50.34%)	10 (6.71%)	38 (25.50%)	27 (18.12%)	0.01 (0.61) ^3^
Respiratory symptom (%)	69 (46.31%)	0 (0%)	21 (14.10%)	48 (32.21%)	0.01 (0.68) ^3^
Right-sided CHF (%)	17 (11.41%)	0 (0%)	1 (0.67%)	16 (10.74%)	0.01 (0.43) ^3^
Parasite burden (1–4)	1.88 ± 0.91	0 ± 0.00	2.13 ± 0.87	2.78 ± 0.76	0.01 (0.79) ^3^
HR (beats per minute)	130.71 ± 24.45	129.63 ± 21.27	132.10 ± 30.51	129.82 ± 18.22	0.848 ^1^
RPADi (%)	33.10 ± 12.07	41.48 ± 4.85	40.32 ± 7.34	20.07 ± 7.14	0.01 (1.77) ^2^
TRPG (mmHg)	24.79 ± 36.92	5.15 ± 3.49	4.08 ± 3.40	59.53 ± 42.10	0.01 (−1.47) ^2^
PT:Ao	1.05 ± 0.20	0.95 ± 0.07	0.92 ± 0.10	1.25 ± 0.16	0.01 (−1.51) ^2^
PV:PA Ratio	0.84 ± 0.34	1.08 ± 0.12	1.05 ± 0.20	0.48 ± 0.21	0.01 (1.76) ^2^
TASPSE	1.45 ± 0.41	1.71 ± 0.32	1.66 ± 0.27	1.06 ± 0.26	0.01 (1.58) ^2^
AT:ET	0.33 ± 0.10	0.39 ± 0.06	0.39 ± 0.07	0.24 ± 0.06	0.01 (1.55) ^2^
E′ (cm/s)	8.94 ± 3.16	10.67 ± 2.26	10.25 ± 2.76	6.45 ± 2.42	0.01 (1.33) ^1^
A′ (cm/s)	10.20 ± 2.98	9.97 ± 1.55	9.79 ± 3.14	10.80 ± 3.38	0.17 ^1^
S (cm/s)	13.44 ± 4.58	14.45 ± 2.36	16.11 ± 3.84	9.85 ± 3.99	0.01 (1.00) ^1^
E′:A′	0.92 ± 0.37	1.08 ± 0.23	1.11 ± 0.37	0.61 ± 0.19	0.01 (1.26) ^1^
HRI-IVCT (ms)	25.01 ± 14.61	15.18 ± 4.81	16.59 ± 6.04	40.45 ± 12.21	0.01 (1.21) ^1^
HRI-IVRT (ms)	47.93 ± 20.26	34.27 ± 6.9	35.95 ± 7.13	69.42 ± 17.13	0.01 (1.73) ^1^
Global TDI	12.99 ± 7.83	15.55 ± 3.75	17.88 ± 7.95	6.05 ± 3.03	0.01 (1.73) ^1^
R-TEI index	0.41 ± 0.19	0.27 ± 0.04	0.30 ± 0.06	0.626 ± 0.17	0.01 (1.80) ^1^

^1^ *p* < 0.01 = ANOVA and Cohen’s d value in brackets. ^2^ *p* < 0.01 = Mann–Whitney/Kruskall–Wallis and Cohen’s d value in brackets. ^3^ *p* < 0.01 = Chi^2^ test and Cramer’s V in brackets.

**Table 2:** Results of simple regression analysis of the cut-off points of TDI measurements by echocardiography to predict the presence of PH using the right pulmonary artery distensibility index (RPADi < 29.5%). R^2^ (coefficient of determination); sensitivity (Se); specificity (Sp); Youden index; area under receiver operating characteristic curve (AUC); confidence interval 95% (CI 95%); tissue Doppler imaging (TDI); global TDI (global TDI index defined as S × E´:A´); isovolumic contraction time indexed to heart rate (HRI-IVCT); isovolumic relaxation time indexed to heart rate (HRI-IVRT); Tei index of right ventricular myocardial performance (R-TEI index).

	R^2^	AUC	CI 95%	Cut-Off Point	Se	Es	*p*-Value	Youden Index (Se + Es − 1)
E′ (cm/s)	0.284	0.863	(0.802, 0.924)	≤8.50	0.818	0.766	0.000	0.584
A′ (cm/s)	0.012	0.572	(0.474, 0.670)	≥11.50	0.455	0.755	0.093	0.210
S (cm/s)	0.281	0.881	(0.816, 0.947)	≤12.50	0.818	0.862	0.000	0.680
E′:A′	0.312	0.938	(0.869, 0.980)	≤0.839	0.873	0.926	0.000	0.798
Global TDI	0.336	0.984	(0.961, 1.000)	≤10.20	0.945	0.989	0.000	0.935
HRI-IVCT (ms)	0.513	0.958	(0.926, 0.990)	≥26.50	0.836	0.957	0.000	0.794
HRI-IVRT (ms)	0.533	0.954	(0.915, 0.994)	≥46.50	0.873	0.989	0.000	0.862
R-TEI index	0.555	0.965	(0.927, 1.000)	≥0.387	0.909	0.989	0.000	0.898

## Data Availability

The raw data supporting the conclusions of this article will be made available by the authors, without undue reservation. Data are contained within the article.
